# Supplementary material for: Stat3 promotes mitochondrial transcription and oxidative respiration during maintenance and induction of naive pluripotency
Source: EMBO J. 2016 Feb 22;35(6):618–34. doi: 10.15252/embj.201592629 (PMC4801951; doi:10.15252/embj.201592629)
Supplement: Supplementary file 1 — Appendix [file EMBJ-35-618-s001.pdf]

Appendix

Stat3 promotes mitochondrial transcription and oxidative respiration during maintenance and induction of naive pluripotency

Elena Carbognin[1], Riccardo M. Betto[1], Maria E. Soriano[4], Austin G. Smith[2,3,5] and Graziano Martello[1,5]

Appendix Figures S1-S10 and figure legends

Appendix Figure S1

A

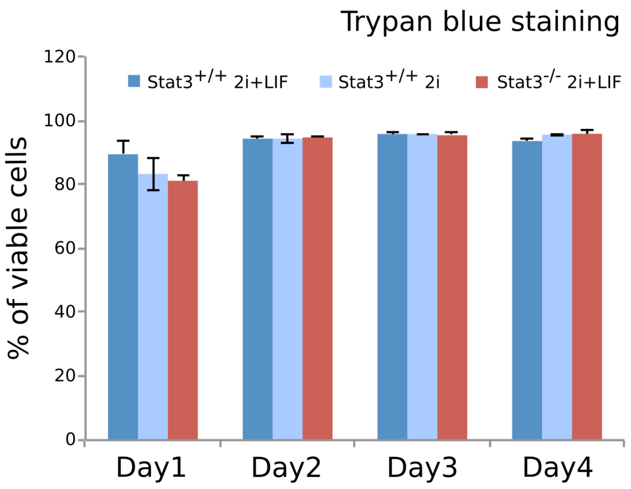

B

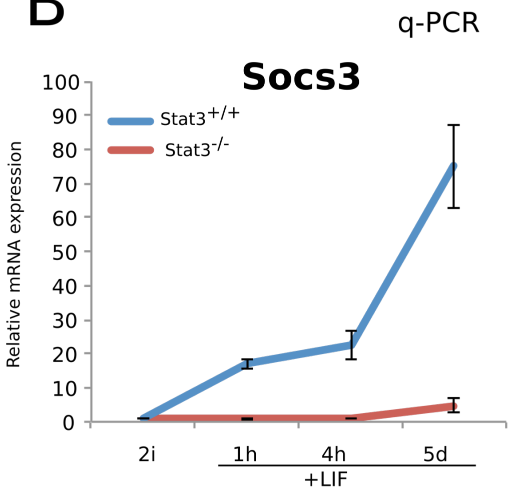

C

Stat3 targets

	2i	+ LIF	+ LIF 24h
**Klf4**	1,00	1,90	2,36
**Stat3**	1,00	2,10	2,32
**Tfcp2l1**	1,00	2,25	2,21

Master regulators

	2i	+ LIF	+ LIF 24h
Tfb1m	1,00	0,86	0,89
Tfb2m	1,00	1,11	0,83
Nrf1	1,00	1,01	1,04
Nfe2l2 /Nrf2	1,00	1,04	0,93
Tfam	1,00	0,78	0,69
Ppargc1a / Pgc-1a	1,00	0,33	0,38
Ppargc1b	1,00	1,07	2,08
Hif1a	1,00	0,88	0,74
Arnt / Hif1 beta	1,00	0,94	0,99
Hif3a	1,00	1,20	1,60

Complex I mito encoded

	2i	+ LIF	+ LIF 24h
mt-Nd1	1,00	1,70	1,60
**mt-Nd2**	1,00	1,82	2,09
mt-Nd3	1,00	2,58	1,23
**mt-Nd4**	1,00	2,26	2,55
mt-Nd4l	1,00	5,44	3,43
**mt-Nd5**	1,00	3,48	3,78
mt-Nd6	1,00	1,25	1,11

5,00	1,00	0,20

Appendix FigureS1:

(**A**) Trypan blue assay on Stat3$^{+/+}$ cells cultured in 2i or 2i+LIF and Stat3$^{-/-}$ cells in 2i+LIF. Equal numbers of cells ware pleated and were cultured in 2i or 2i+LIF conditions. At regular intervals of 24h the cells were detached, treated with Trypan blue (a specific dye for dead cells) and counted. Histogram shows the percentage of viable cells in the three conditions. No significant difference in the percentage of viable cells is observed. Mean and s.e.m of two independent experiments is shown.

(**B**) Gene expression analysis of Stat3$^{+/+}$ and Stat3$^{-/-}$ cells cultured in 2i and subjected to LIF treatment of 1h, 4h and 5days. Note that Socs3 expression is not induced by LIF in Stat3$^{-/-}$ cells.

(**C**) Gene expression analysis from RNA sequencing data of Stat3$^{+/+}$ cells cultured in 2i and treated for 1 hour or 24 hours with LIF. Fold change of nuclear encoded master regulators of mitochondrial transcription are shown. Genes that show an upregulation upon LIF treatment are indicated in blue, genes downregulated are indicated in red. Data were normalized to untreated sample (2i). The known nuclear Stat3 targets Klf4, Stat3 itself and Tfcp2l1 (left), as well as the mitochondrially encoded subunits of Complex I (right), are shown as a reference.

Appendix Figure S2

A

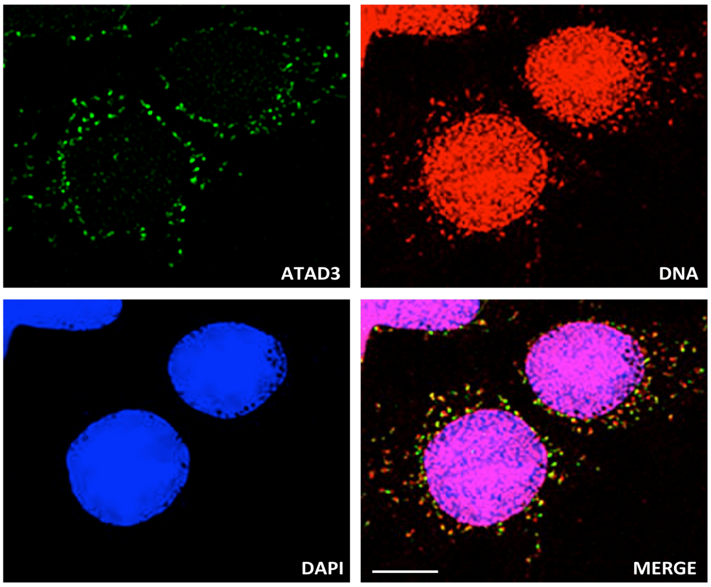

Appendix Figure S2:

(**A**) Representative confocal images of MLS-Stat3 cells stained with anti-ATAD3 (top left) and anti-DNA (top right) antibody. Merge image shows co-localization between ATAD3 and the mitochondrial DNA (R=0.67) confirming that ATAD3 is a good nucleoid marker in murine ES cells (bottom right). Scale bar, 10μm.

Appendix Figure S3

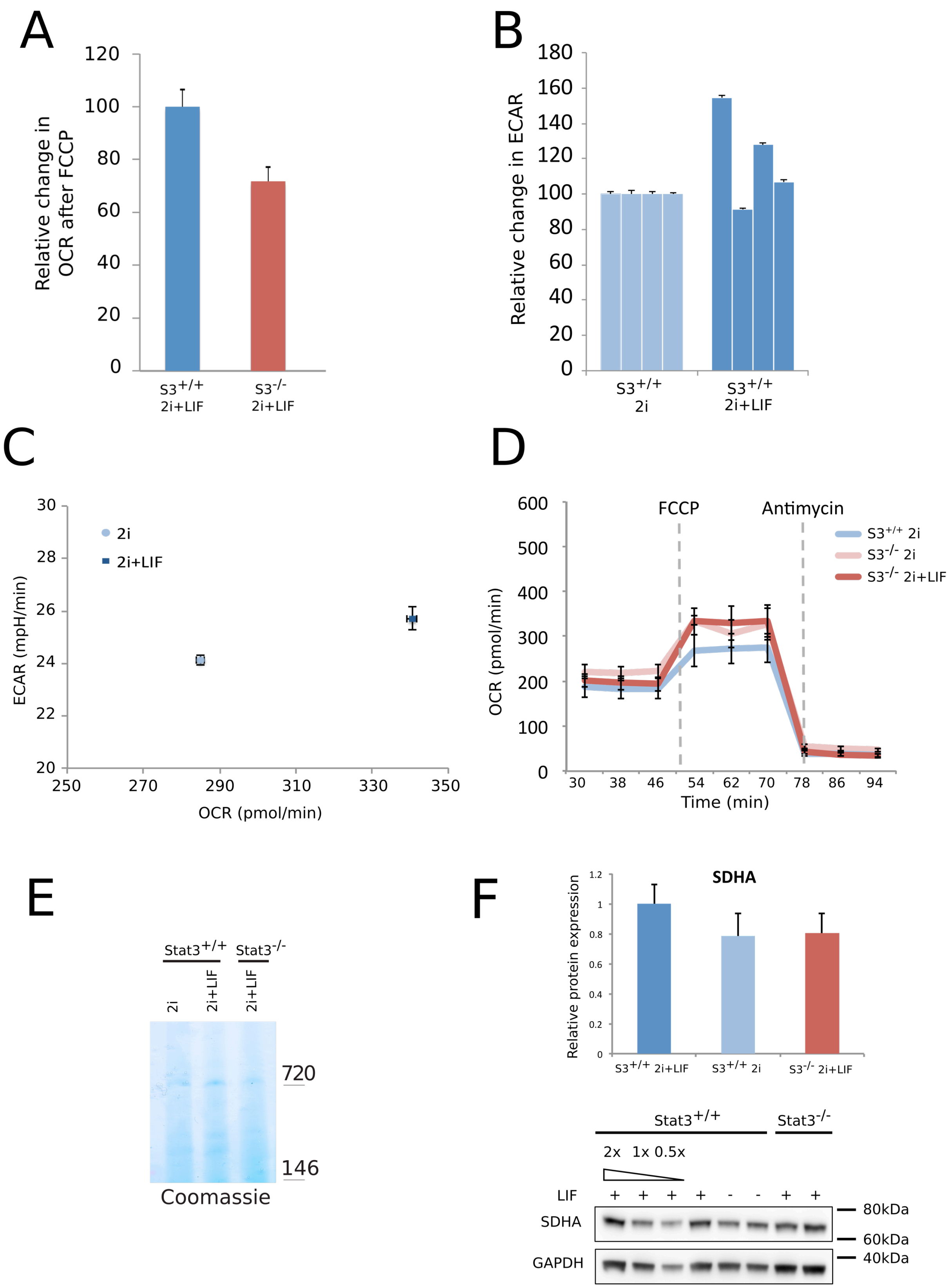

Appendix FigureS3:

(**A**) Relative changes in oxygen consumption after 200nM FCCP treatment of $Stat3^{+/+}$ (blue bar) and $Stat3^{-/-}$ cells (red bars) cultured in 2i+LIF. Mean and s.e.m. of >4 technical replicates are shown.

(**B**) Relative changes in glycolysis measured by extracellular acidification rate (ECAR) of $Stat3^{+/+}$ cells cultured in 2i (light blue bars) or 2i with LIF (dark blue bars). Basal levels of ECAR were measured. Mean and s.e.m. of 5 technical replicates for 4 independent experiments is shown.

(**C**) Basal OCR and ECAR measurements of $Stat3^{+/+}$ cells cultured in 2i and 2i+LIF. LIF enhances basal levels of OCR and does not reduce the basal levels of ECAR. Mean and s.e.m. of three technical replicates is shown.

(**D**) Oxygen consumption rate (OCR) measured by SeaHorse Extracellular Flux assay of $Stat3^{+/+}$ in 2i (light blue) and $Stat3^{-/-}$ cells maintained in 2i or with LIF (light and dark red respectively). No significant changes among the 3 samples were detected. Mean and s.e.m. of 5 technical replicates is shown.

(**E**) Comassie staining of NBGE gel served as a loading control for figure 3E.

(**F**) Bottom: Western blot for SDHA (a subunit of ComplexII) of $Stat3^{+/+}$ cells cultured in 2i or 2i+LIF and $Stat3^{-/-}$ cells in 2i+LIF. GAPDH was used as an endogenous control. Top: quantification of the Western blot. Levels of SDHA appear slightly lower in absence of LIF or in the absence of Stat3, but such differences are not statistically significant (p-value >0.05, unpaired t-test).

Appendix Figure S4

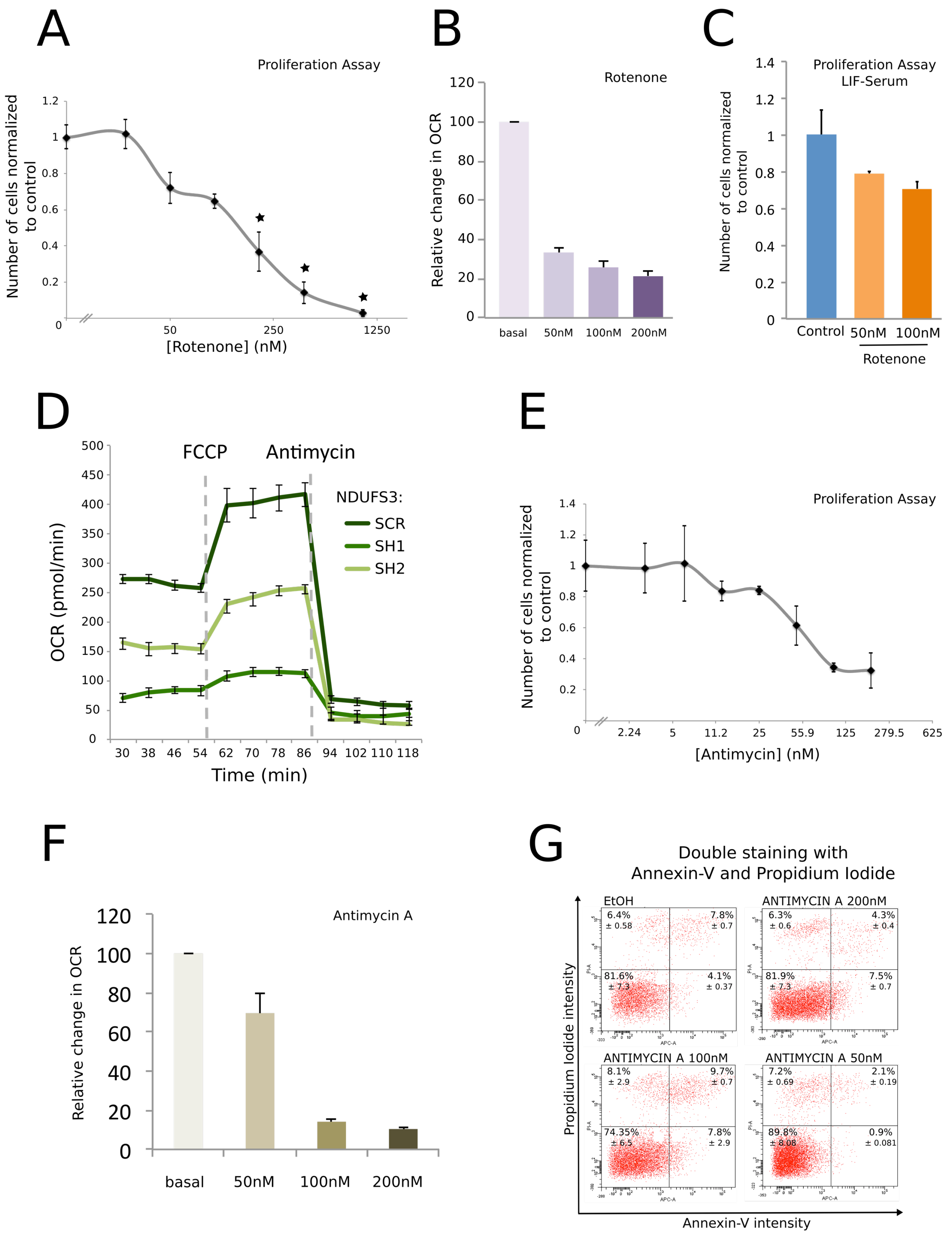

Appendix FigureS4:

(**A**) Titration of Rotenone effects on proliferation of mES cells. Stat3$^{+/+}$ cells were cultured for 48h in presence of increasing doses of Rotenone. Scores were normalized to control cells treated with DMSO. The x axis shows the concentration of Rotenone on a logarithmic scale. Only in the doses indicated by stars (>200nM) we observed overt cell death, confirmed also by PI/Annexin-V staining in Figure 4A. Mean and st.dev of 3 independent experiments.

(**B**) Relative changes in oxygen consumption of Stat3$^{+/+}$ cells after treatment with increasing doses of Rotenone. Basal levels of OCR were measured before Rotenone treatment and used to calculate the relative change in OCR. Note that 50nM Rotenone is sufficient to reduce mitochondrial respiration of 70%. Mean and s.e.m. of 5 biological replicates is shown.

(**C**) Proliferation assay of Stat3$^{+/+}$ cells cultured in Serum media in presence of LIF. Cells were seeded and counted after 48hours treatment with 50nM (light orange) or 100nM (dark orange) Rotenone. Scores were normalized to untreated control. Mean and s.e.m. of 3 independent experiments is shown.

(**D**) Oxygen consumption rate (OCR) measured by SeaHorse Extracellular Flux assay of Stat3$^{+/+}$ cultured in 2i+LIF and transfected with control shRNA and two shRNAs for NDUFS3. Note that knockdown of the ComplexI subunit significantly affects respiration. Mean and s.e.m. of 5 technical replicates is shown.

(**E**) Titration of Antimycin effects on proliferation of mES cells. Stat3$^{+/+}$ cells were cultured for 48h in presence of increasing doses of Antimycin. The x axis shows the concentration of Antimycin on a logarithmic scale. No overt cell death was observed, as confirmed in Appendix Figure S4G. Scores were normalized to control treated with Ethanol. Mean and s.e.m. of 2 independent experiments.

(**F**) Relative changes in oxygen consumption of Stat3$^{+/+}$ cells after treatment with increasing doses of Antimycin. Basal levels of OCR were measured before Antimycin treatment and used to calculate the relative change in OCR. Mean and s.e.m. of 5 biological replicates is shown.

(**G**) Flow cytometry analysis after double staining with Annexin-V and Propidium Iodide in Stat3$^{+/+}$ cells. Viable unstained cells are indicated on bottom left quadrants, early apoptotic cells (Annexin V-FITC positive) on bottom right quadrants and late apoptotic and/or necrotic cells (Annexin V-FITC and Propidium Podide positive) on top right quadrants. Cells were treated with increasing concentrations of Antimycin from 50nM to 200nM for 48 hours. In each quadrant the mean and s.e.m. of 3 independent experiments is indicated.

Appendix Figure S5

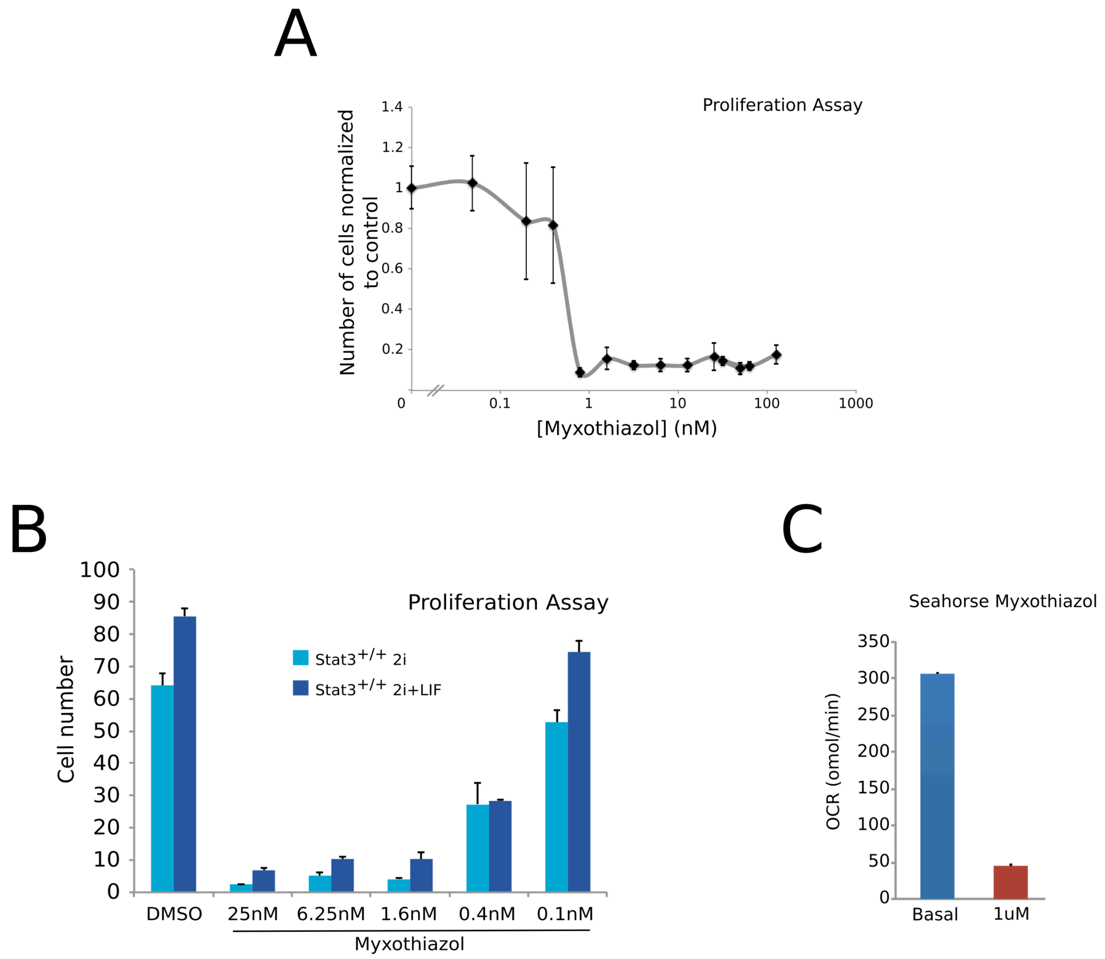

Appendix FigureS5:

(**A**) Titration of Myxothiazol effects on proliferation of mES cells. Stat3$^{+/+}$ cells were cultured for 48h in presence of increasing doses of Myxothiazol. The x axis shows the concentration of Myxothiazol on a logarithmic scale. No overt cell death was observed. Scores were normalized to control treated with DMSO. Mean and s.d. of 2 independent experiments.

(**B**) Proliferation assay of Stat3$^{+/+}$ cells cultured in 2i (light blue bars) or in 2i+LIF (dark blue bars). Cells were seeded and counted after 48hours treatment with decreasing doses of Myxothiazol (a know inhibitor of ComplexIII) ranging from 25nM to 0.1nM. Note that doses from 25nM to 0.4nM reduce proliferation. Scores were normalized to control cells treated with DMSO. Mean and s.e.m. of three independent experiments are shown.

(**C**) Oxygen consumption rate (OCR) measured by SeaHorse Extracellular Flux assay of Stat3+/+ cultured in 2i+LIF (basal, blue bar) and after treatment with 1uM Myxothiazol (red bar). Mean and s.e.m. of 5 technical replicates is shown.

Appendix Figure S6

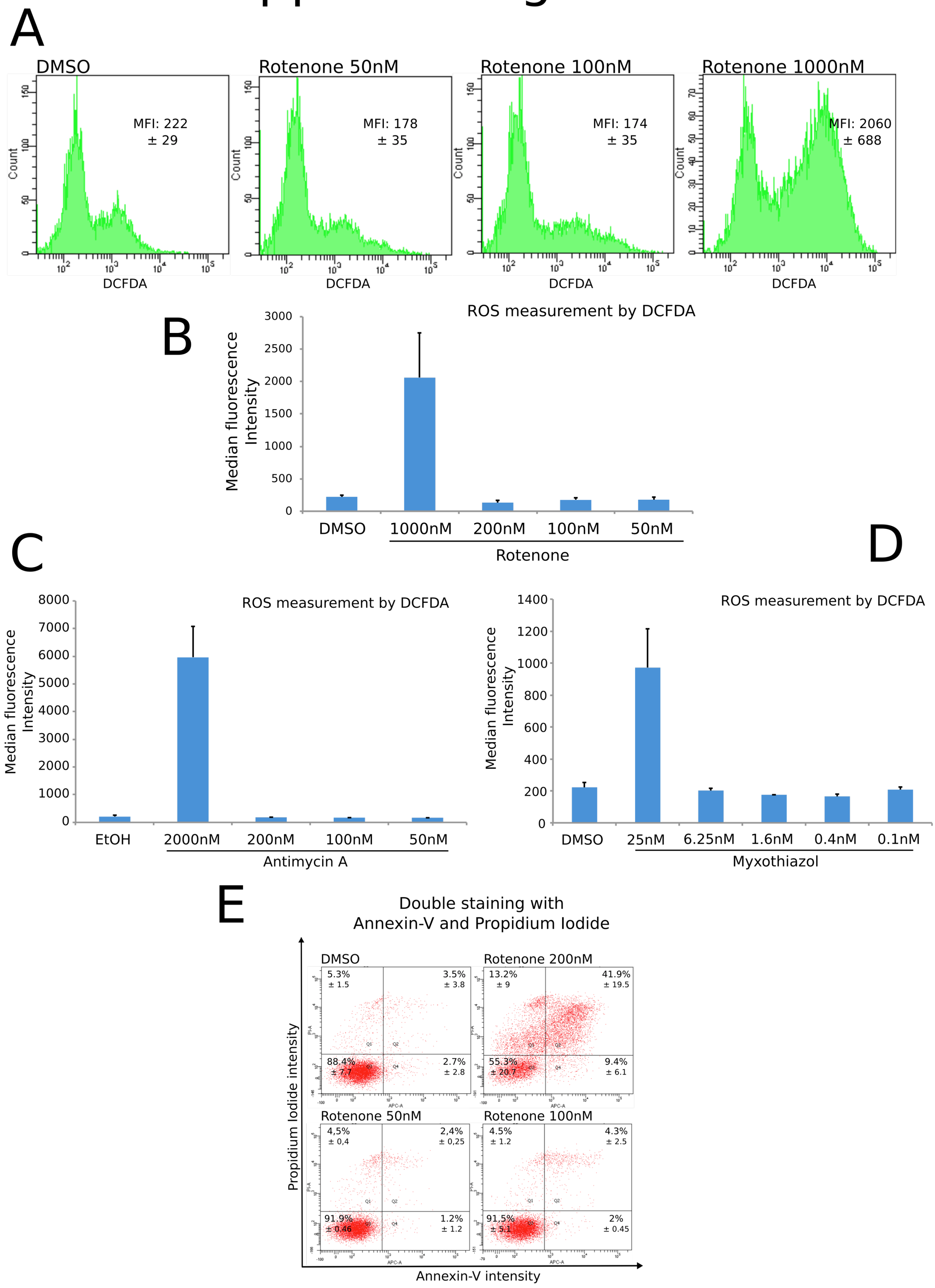

Appendix FigureS6:

(A) Representative plots of flow cytometry analysis of Stat3$^{+/+}$ cells treated for 48 hours with 50nM, 100nM and 1000nM of Rotenone and stained with dichlorofluorescin diacetate (DCFDA), in order to detect ROS production in live cells. Cells treated with DMSO were used as a negative control. Note that only treatment with 1000nM of Rotenone induces ROS production. All other doses, which have been used in our biological assays, do not show any effect.

(B) Histogram showing quantification of median fluorescence intensity of Stat3$^{+/+}$ treated with increasing doses of Rotenone and stained with DCFDA (see also Appendix Figure S6A).

(C) Histogram showing quantification of median fluorescence intensity of Stat3$^{+/+}$ treated for 48hours with decreasing doses of Antimycin A and stained with DCFDA. Cells treated with Ethanol were used as negative control. Note that only 2000nM Antimycin A treatment induces ROS production. All other doses, which have been used in our biological assays, do not show any effect.

(D) Histogram showing quantification of median fluorescence intensity of Stat3$^{+/+}$ treated for 48hours with decreasing doses of Myxothiazol and stained with DCFDA. Cells treated with DMSO were used as negative control. Note that only 25nM Myxothiazol treatment induces ROS production. All other doses, which have been used in our biological assays, do not show any effect.

(E) Flow cytometry analysis after double staining with Annexin-V and Propidium Iodide in Stat3$^{+/+}$ cells. Viable unstained cells are indicated on bottom left quadrants, early apoptotic cells (Annexin V-FITC positive) on bottom right quadrants and late apoptotic and/or necrotic cells (Annexin V-FITC and Propidium Podide positive) on top right quadrants. Cells were treated with increasing concentrations of Rotenone from 50nM to 200nM for 4 passages. In each quadrant the mean and s.e.m. of 2 independent experiments is indicated.

Appendix Figure S7

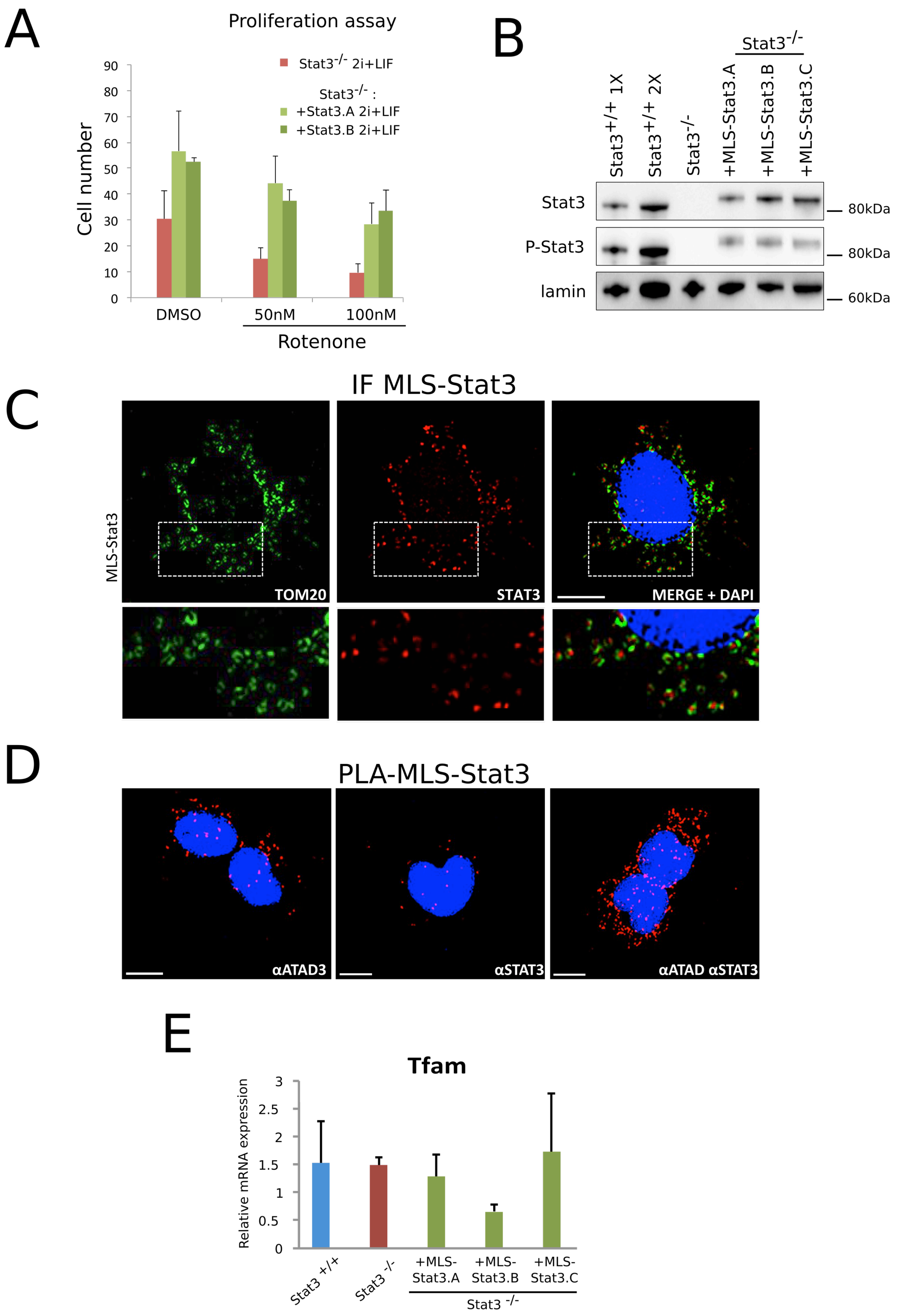

Appendix FigureS7:

(**A**) (Proliferation assay of Stat3$^{-/-}$ cells and Stat3.A/B rescue clones cultured in the presence of LIF and 50nM and 100nM of rotenone. Cells were seeded and treated with DMSO or rotenone at the indicated concentrations for 48hours. Note that rescue clones are less affected by rotenone than Stat3$^{-/-}$ cells. Mean and s.e.m. of three biological replicates.

(**B**) Western blot of Stat3$^{+/+}$ cells, Stat3$^{-/-}$ cells and three clones of Stat3-/- cells transfected with a construct expressing Stat3 cDNA fused to a Mitochondrial Localization Signal (MLS), cultured in the presence of LIF. Note that MLS-Stat3 protein levels in all three clones are comparable to the endogenous levels and that a fraction of the fusion protein is phosphorylated (Tyr705) in the presence of LIF. LaminB served as a loading control.

(**C**) Representative confocal images of MLS-Stat3 cells stained with anti-TOM20, marking external mitochondrial membrane (left) and anti-Stat3 (middle) antibody. Merge image (right) shows that Stat3 and TOM20 expression is adjacent but non overlapping, suggesting that Stat3 does not localise to the external mitochondrial membrane (R=0.35). Scale bar, 10μm.

(**D**) Representative confocal images of MLS-Stat3 cells subjected to proximity-ligation-assay (PLA) by using anti-Stat3 and anti-ATAD3 antibodies. Only when the two proteins are close to each other an enzymatic reaction takes place, producing discrete fluorescent red dots in the nanometer range. Anti-Stat3 or anti-ATAD3 alone were used to asses the assay specificity. DAPI serves as a nuclear counterstain. Scale bar, 10μm.

(**E**) Gene expression analysis of Stat3$^{+/+}$, Stat3$^{-/-}$ and three MLS-Stat3 clones cultured in 2i+LIF. Data show no differences in expression of Tfam, a well known coactivator of mitochondrial transcription. Mean and st.dev. of two biological replicates are shown.

Appendix Figure S8

A

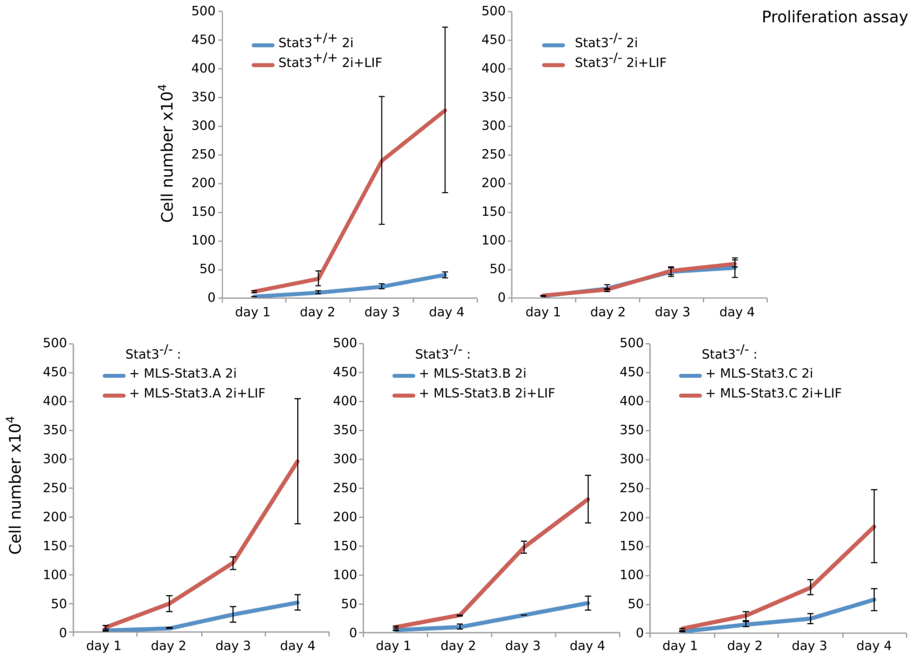

B

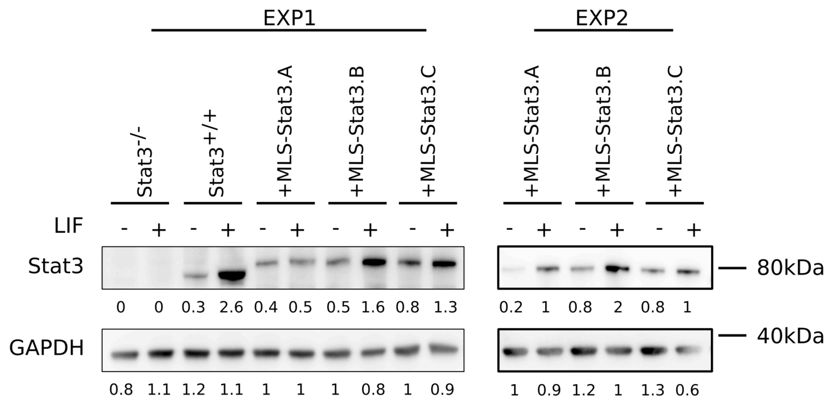

Appendix FigureS8:

(**A**) Proliferation assay of $Stat3^{+/+}$, $Stat3^{-/-}$ cells and three MLS-Stat3 clones cultured in 2i or 2i+LIF. Cells were seeded and scored for four days. Mean and s.e.m. of two technical replicates of a representative experiment are shown.

(**B**) Western blot of two independent experiments on $Stat3^{+/+}$ cells, $Stat3^{-/-}$ cells and three MLS-Stat3 clones cultured in 2i or 2i+LIF. Note that Stat3 protein levels are increased by LIF in all cell lines. GAPDH was used as a loading control.

Appendix Figure S9

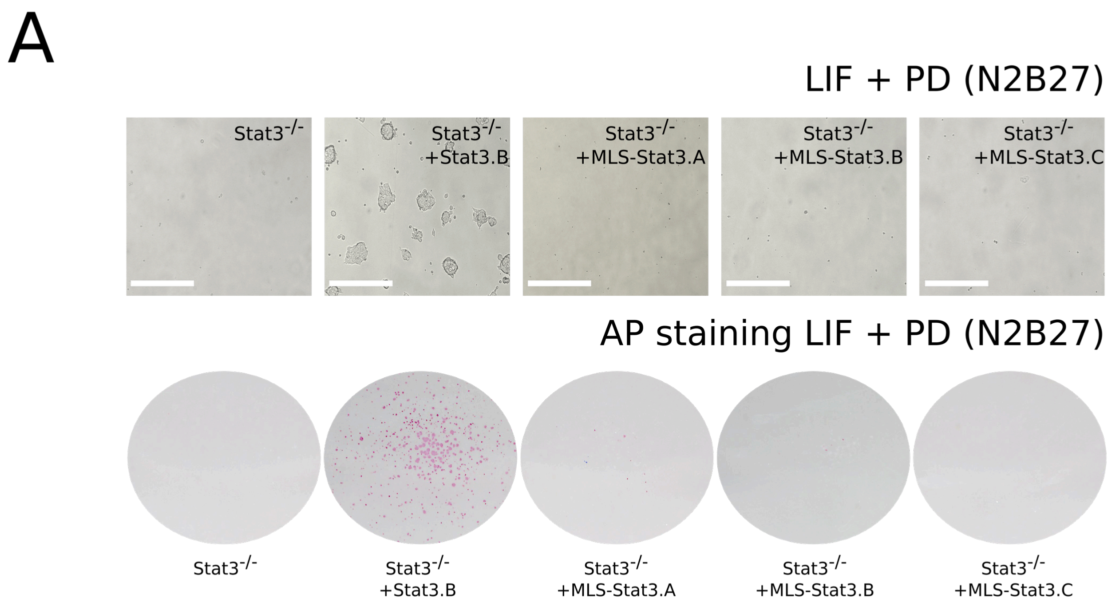

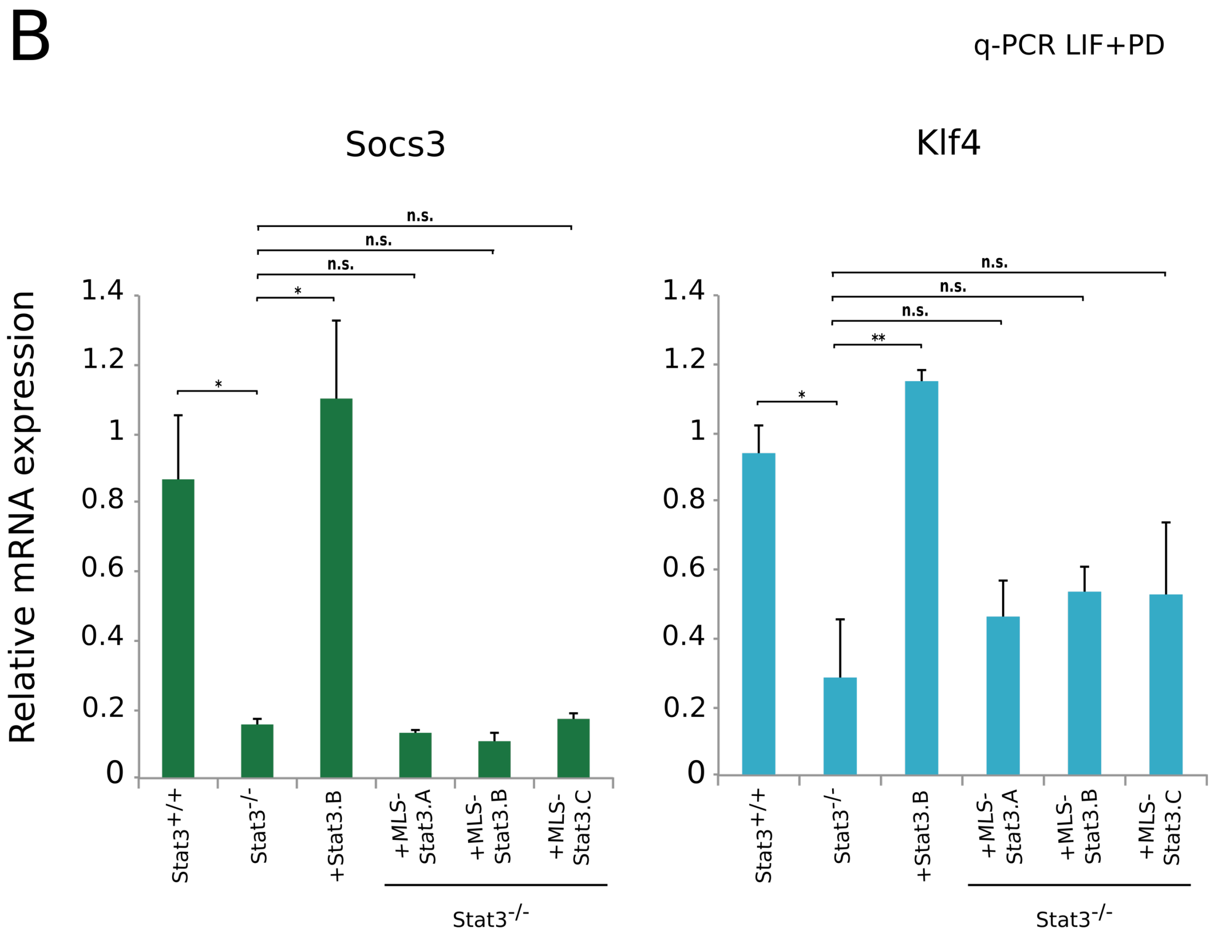

Appendix Figure S9:

(**A**) Top: Bright field images of $Stat3^{-/-}$ cells, Stat3 rescue clone (Stat3.B) and three MLS-Stat3 clones cultured in LIF and PD for 4 passages. Note that after 4 passages in LIF and PD medium, $Stat3^{-/-}$ cells and MLS-Stat3 clones, but not Stat3 rescue cells, collapsed. Bottom: AP staining at passage 4 of $Stat3^{-/-}$ cells, Stat3 rescue clone (Stat3.B) and three MLS-Stat3 clones cultured in LIF and PD medium. Scale bar, 50μm.

(**B**) Gene expression analysis of the two Stat3 direct targets Socs3 (left) and Klf4 (right) of the indicated cell lines cultured in LIF+PD for 3 passages. $Stat3^{-/-}$ cells were analysed after one passage because they differentiated and collapsed at passage 2. Mean and s.d. of two independent experiments are shown.

Appendix Figure S10

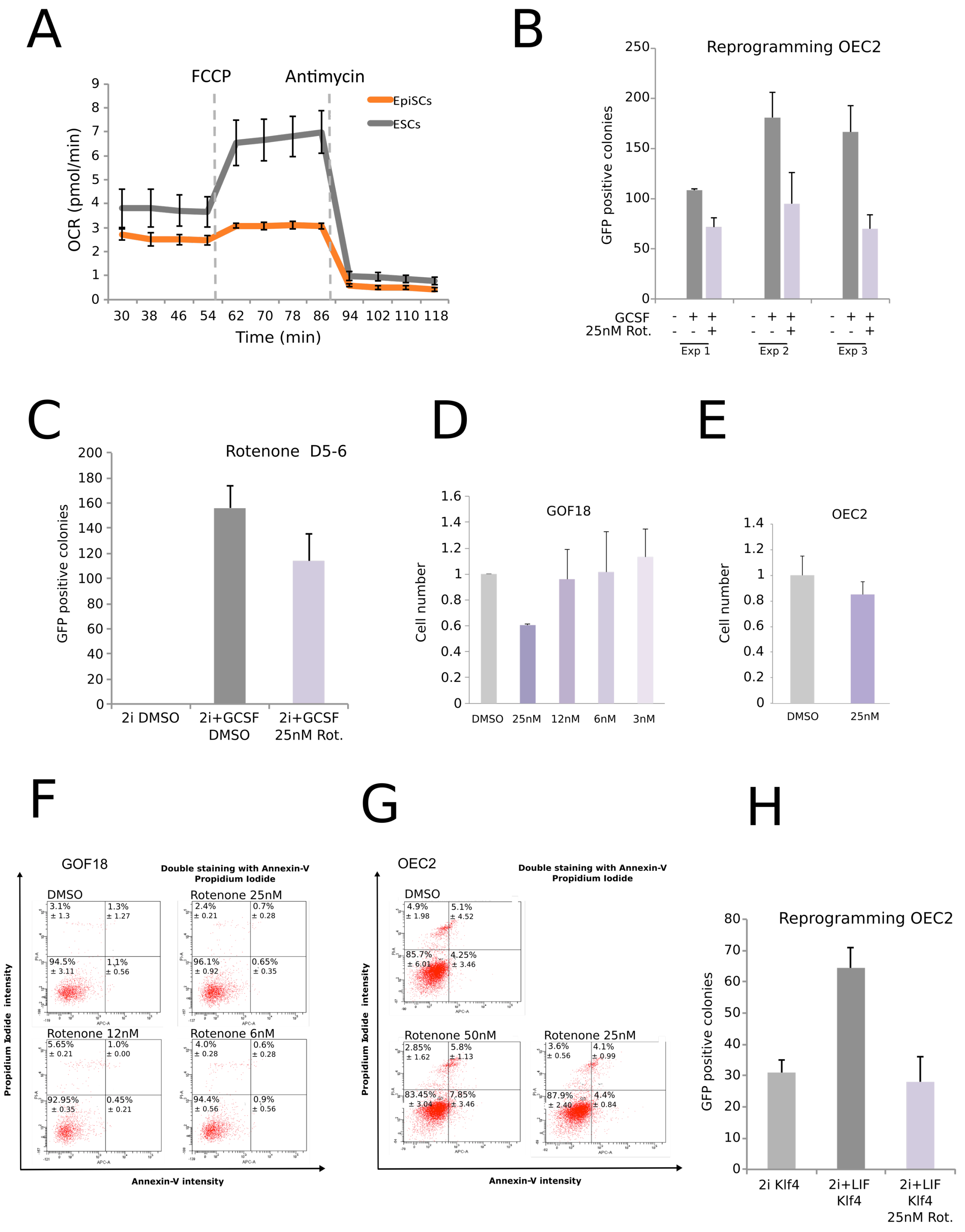

Appendix FigureS10:

(**A**) Oxygen consumption rate (OCR) measured by SeaHorse Extracellular Flux assay of $Stat3^{+/+}$ ES cells maintained in 2i+LIF and GOF18 EpiSCs maintained in FGF+Activin in chemically defined media N2B27 in the absence of feeders. 200nM FCCP treatment resulted in higher OCR increase in ES cells compared to EpiS cells, showing a higher level of maximal mitochondrial electron transport chain (ETC) activity in ES cells. Injection of 200nM Antimycin shows similar non-mitochondrial respiration rates for both cell types. Mean and s.e.m. of >3 technical replicates is shown.

(**B**) Quantification of Oct4-GFP positive iPS colonies at day7 of reprogramming generated from OEC2 Y118 EpiSCs subjected to 48hours treatment with GCSF and 25nM of Rotenone. Note that Rotenone treatment reduces the number of iPS colonies generated. Three independent experiments are shown. Mean and s.e.m of two technical replicates is indicated.

(**C**) Number of Oct4-GFP positive colonies generated from OEC2 Y118 EpiSCs at day8 of reprogramming upon 48hours treatment with Rotenone at day5-6. Note that Rotenone treatment given at day5-6 does not reduce the number of iPS colonies. Mean and s.e.m of two technical replicates is shown.

(**D**) Proliferation assay of GOF18 EpiSCs line cultured in FGF+Activin media and treated for 48 hours with DMSO or decreasing doses of Rotenone. Note that doses ranging from 3nM to 12nM do not reduce cell number. Data were normalized to samples treated with DMSO and mean and s.e.m of 2 independent experiments is shown.

(**E**) Proliferation assay of OEC2 Y118 EpiSCs line cultured in FGF+Activin media and treated for 48 hours with DMSO or 25nM Rotenone. Data were normalized to samples treated with DMSO and mean and s.e.m of 2 technical replicates is shown.

(**F**) Flow cytometry analysis after double staining with Annexin-V and Propidium Iodide in GOF18 EpiSCs. Viable unstained cells are indicated on bottom left quadrants, early apoptotic cells (Annexin V-FITC positive) on bottom right quadrants and late apoptotic and/or necrotic cells (Annexin V-FITC and Propidium Podide positive) on top right quadrants. Cells were treated with DMSO or decreasing concentrations of Rotenone from 25nM to 6nM for 48 hours. In each quadrant mean and s.e.m. of 2 technical replicates is indicated.

(**G**) Flow cytometry analysis after double staining with Annexin-V and Propidium Iodide in OEC2 EpiS cells. Cells were treated with DMSO or two doses of Rotenone (50nM and 25nM) for 48 hours. In each quadrant mean and s.e.m. of 2 technical replicates is indicated.

(**H**) Number of iPS colonies generated from OEC2 Y118 EpiSCs transfected with a piggyBac vector containing Klf4 and treated with LIF for 48hours and Rotenone as indicated. Mean and s.e.m. of two independent experiment is shown.
