# Supplementary figures and images for: Stat3 promotes mitochondrial transcription and oxidative respiration during maintenance and induction of naive pluripotency
Source: EMBO J. 2016 Feb 22;35(6):618–34. doi: 10.15252/embj.201592629 (PMC4801951; doi:10.15252/embj.201592629)

SOURCE DATA – Appendix Figure S8B

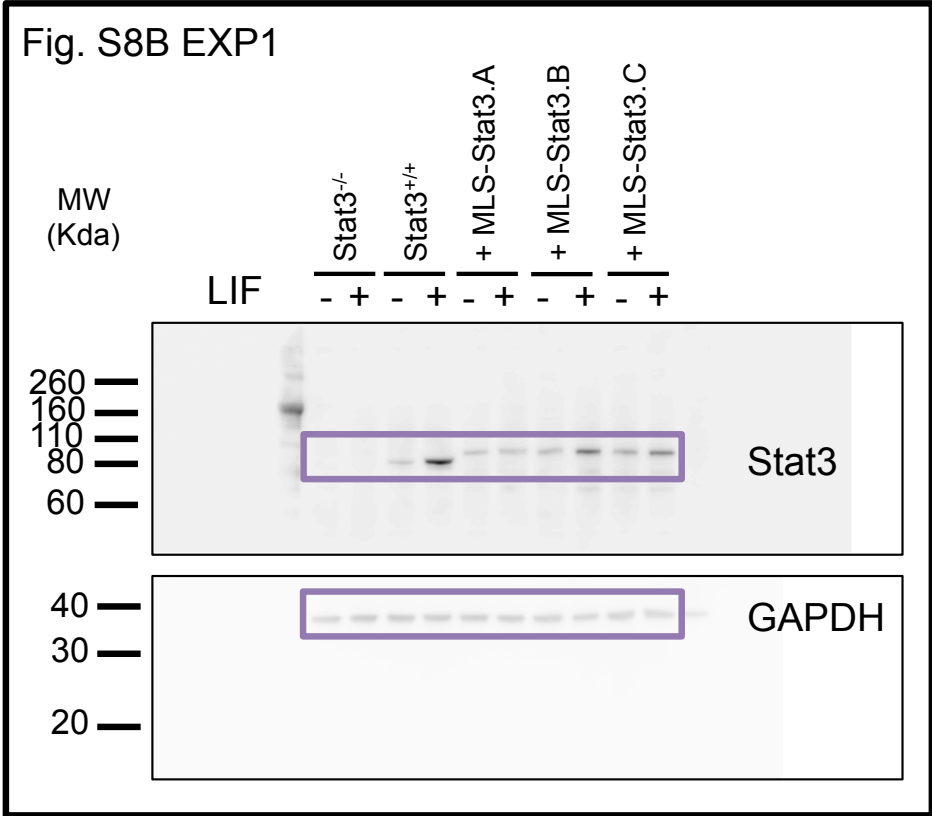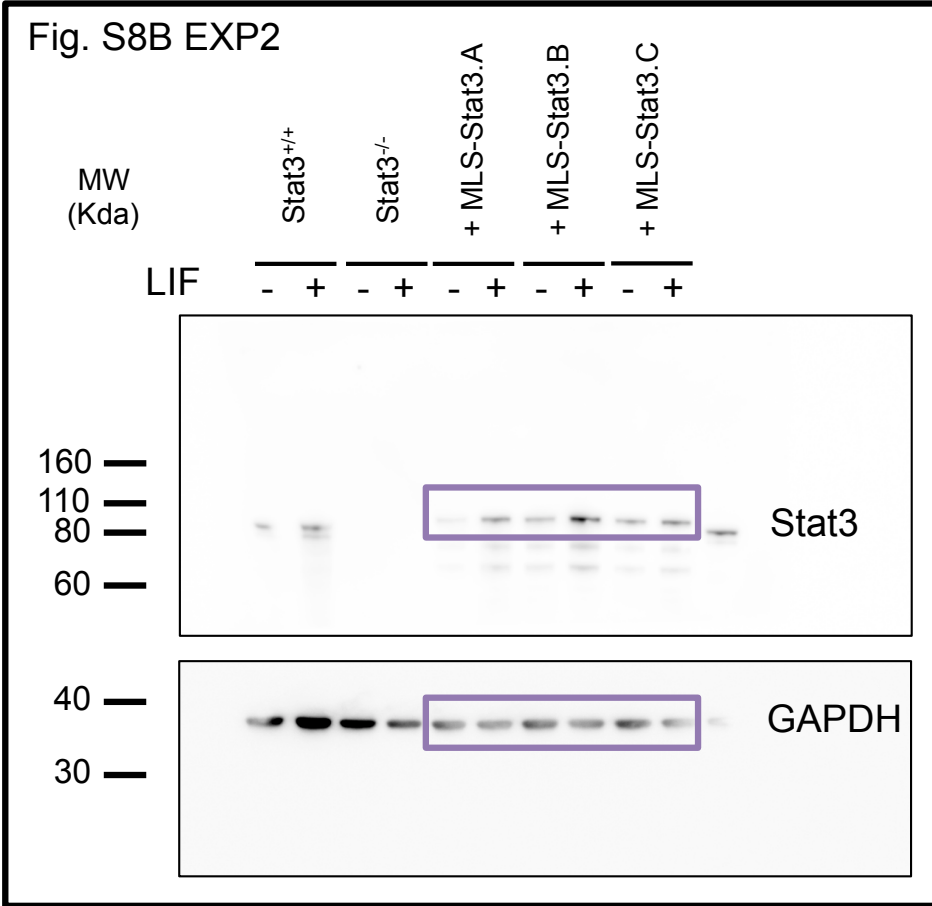

Supplement: Supplementary file 2 — Source Data for Appendix [file EMBJ-35-618-s004.zip › SOURCE DATA Appendix Figure S8B.pdf]

SOURCE DATA – Appendix Figure S3F

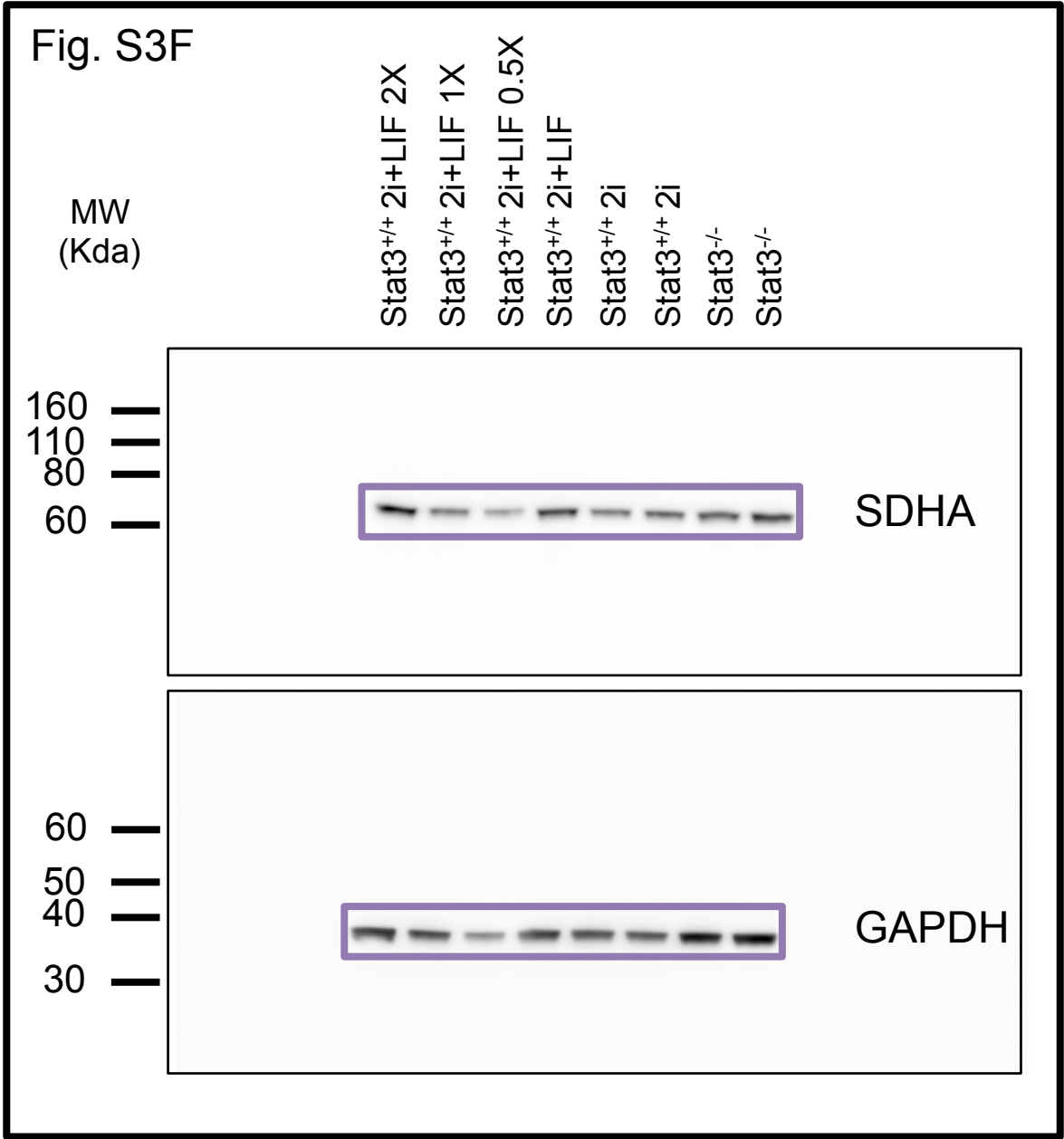

Supplement: Supplementary file 2 — Source Data for Appendix [file EMBJ-35-618-s004.zip › SOURCE DATA Appendix Figure S3F.pdf]

**SOURCE DATA – Appendix Figure S7B**

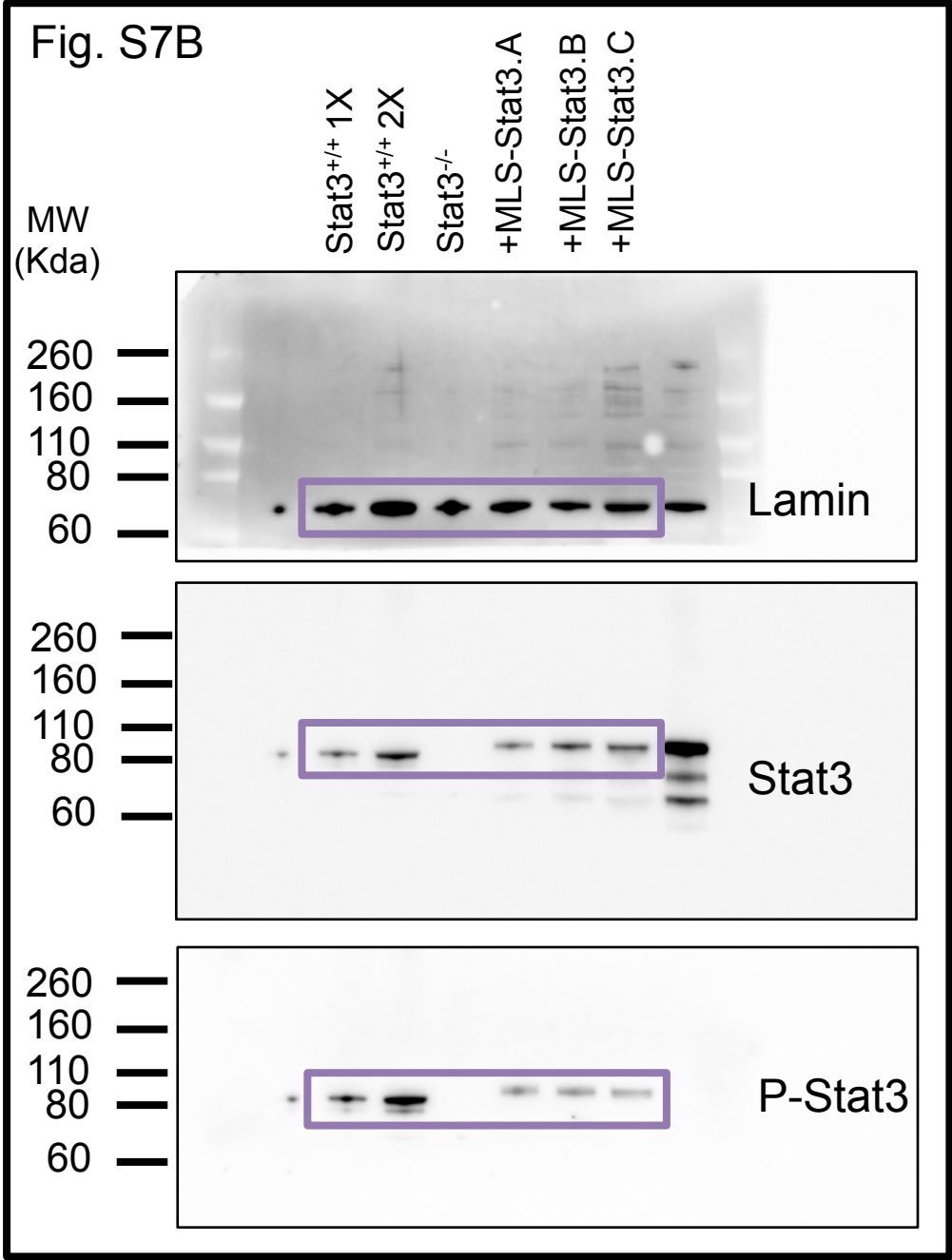

Supplement: Supplementary file 2 — Source Data for Appendix [file EMBJ-35-618-s004.zip › SOURCE DATA Appendix Figure S7B.pdf]

### SOURCE DATA – Figure 3D

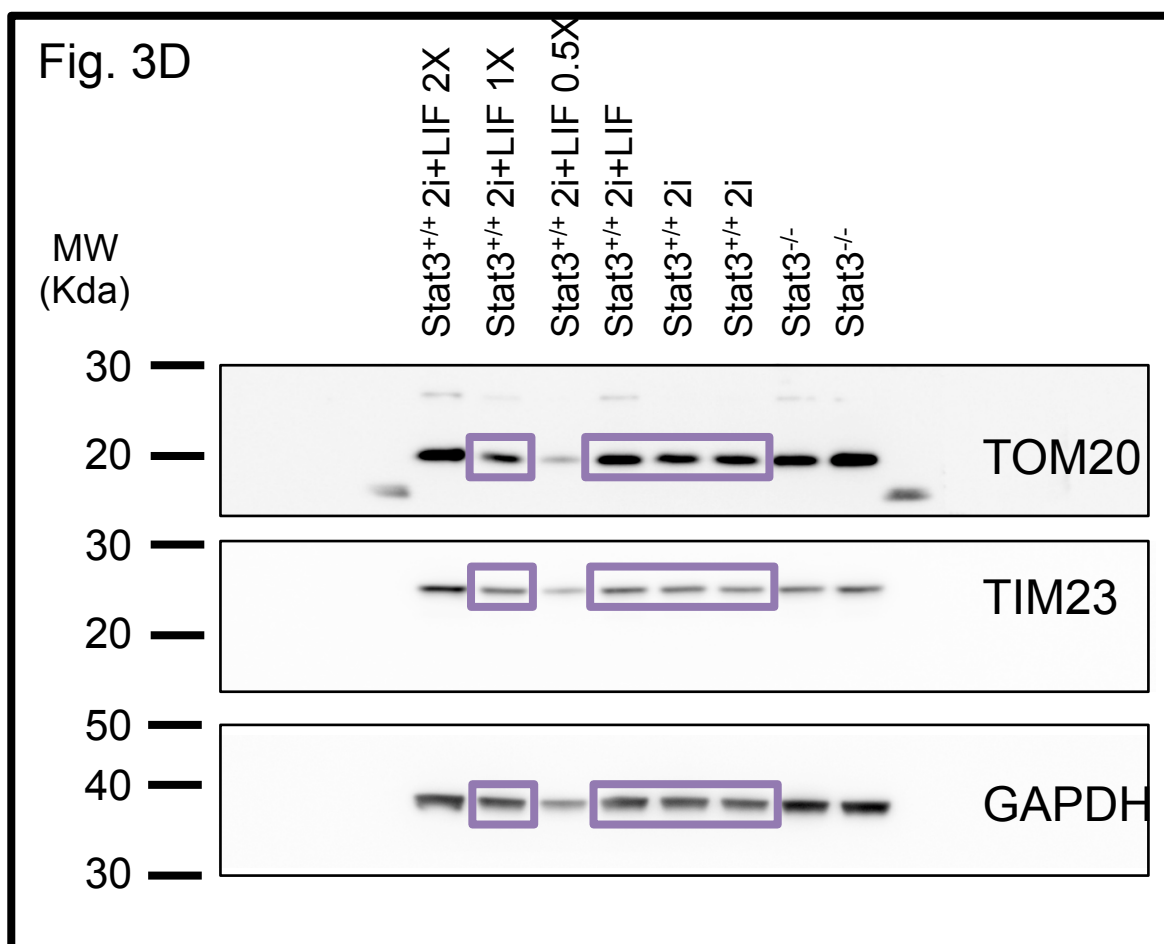

Supplement: Supplementary file 4 — Source Data for Figure 3D [file EMBJ-35-618-s002.pdf]

SOURCE DATA – Figure 5B

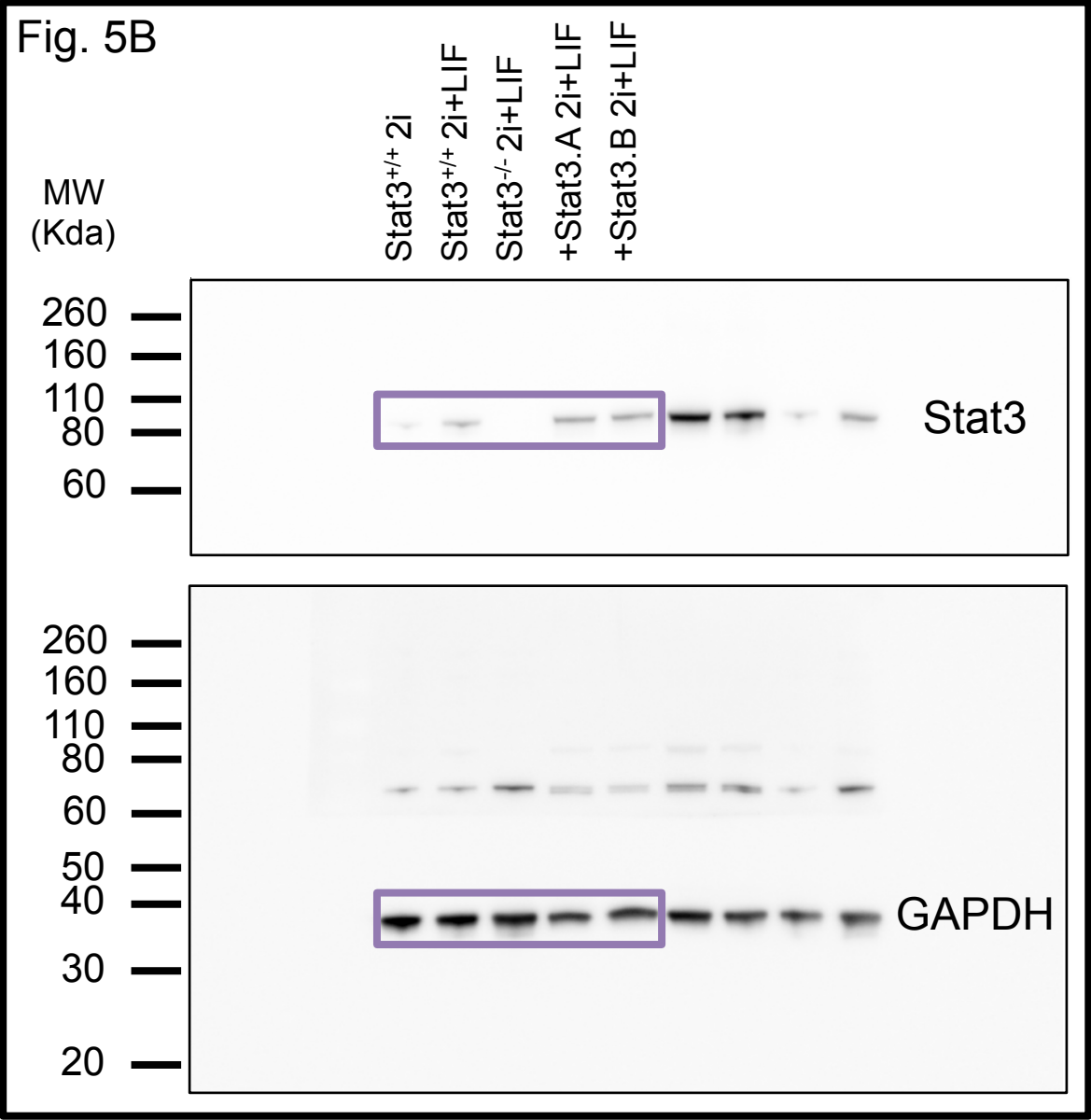

Supplement: Supplementary file 5 — Source Data for Figure 5 [file EMBJ-35-618-s003.zip › embj201592629-sup-0003-SDataFig5B.pdf]

SOURCE DATA – Figure 5E

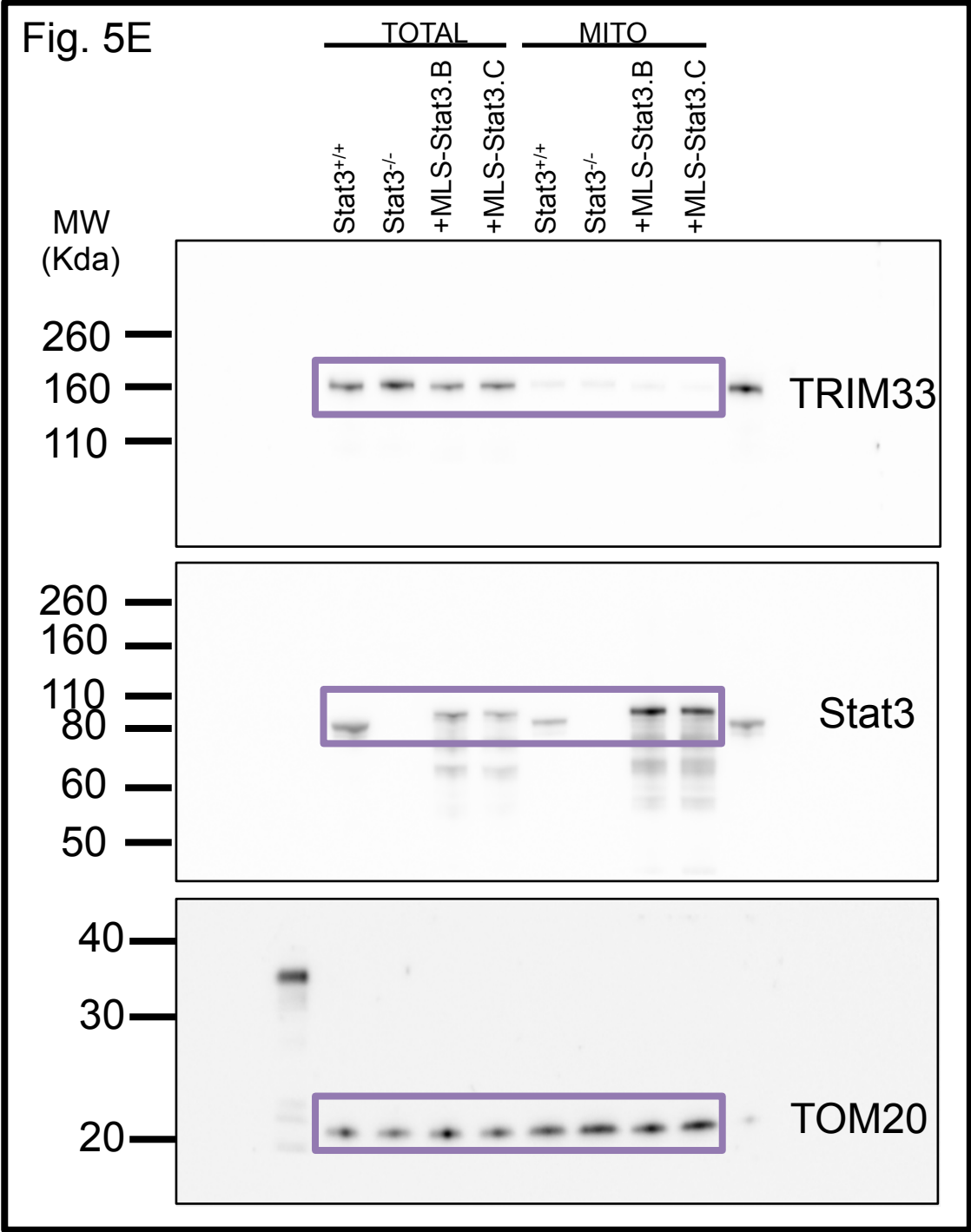

Supplement: Supplementary file 5 — Source Data for Figure 5 [file EMBJ-35-618-s003.zip › embj201592629-sup-0004-SDataFig5E.pdf]
